# Fitness and health-related quality of life dimensions in community-dwelling middle aged and older adults

**DOI:** 10.1186/1477-7525-9-117

**Published:** 2011-12-22

**Authors:** Pedro R Olivares, Narcis Gusi, Josue Prieto, Miguel A Hernandez-Mocholi

**Affiliations:** 1 Faculty of Sports Sciences, University of Extremadura, 10003 Caceres, Spain

## Abstract

**Background:**

The aim of the present study was to identify the physical fitness (PF) tests of a multi-component battery more related to the perception of problems in each dimension of the health-related quality of life (HRQoL) assessed by the EuroQol 5 dimensions 3 level questionnaire (EQ-5D-3L) in community-dwelling middle-aged and older adults

**Methods:**

A cross-sectional study was conducted with 7104 participants (6243 females and 861 males aged 50-99 years) who were recruited in the framework of the Exercise Looks After You Program, which is a public health program designed to promote physical activity (PA) in community-dwelling middle-aged and older adults. Participants were assessed by the EQ-5D-3L questionnaire and a battery of fitness tests. The responses to each EQ-5D-3L dimension were collapsed into a two-tier variable consisting of «perceive problems» and «do not perceive problems». Correlation coefficients for the relationships between the HRQoL variables, between the PF variables, and between the HRQoL and PF variables were obtained. Two logistic regression models, one adjusted and one unadjusted, were developed for each EQ-5D-3L dimension.

**Results:**

There were significant correlations between all variables except anxiety/depression and the back scratch test. The PF tests that correlated best with the HRQoL dimensions were the Timed Up-and-Go Test (TUG) and the 6-min walk; pain/discomfort and anxiety/depression correlated less well. All PF tests correlated, especially the TUG and 6-min walk tests. Unadjusted logistic models showed significant goodness of fit for the mobility and pain/discomfort dimensions only. Adjusted logistic models showed significant goodness of fit for all dimensions when the following potential confounding variables were included: age, gender, weekly level of PA, smoking and alcohol habits, body mass index, and educational level. For all dimensions, the highest odds ratios for the association with PF tests were with the TUG; this was observed with both the unadjusted and adjusted models.

**Conclusions:**

The perception of problems, as measured by the EQ-5D-3L dimensions, was associated with a lower level of fitness, particularly for those dimensions that relate more closely to physical components. The PF tests that associated most closely with the perception of problems in the HRQoL dimensions were the TUG and the 6-min walk. This information will aid the design and assessment of PA programs that aim to improve HRQoL.

## Background

A major goal of health-enhancing physical activity (PA) is to preserve or improve health-related quality of life (HRQoL). PA and physical fitness (PF) are closely related in that PF is mainly, although not entirely, determined by PA patterns [1]. Associations between PA and HRQoL have been reported in some studies but associations between PF and HRQoL have not been examined sufficiently.

The scarce literature that is available shows that people with better PF scores usually reports higher percentile scores for several HRQoL domains that are measured by the Short Form 36 Questionnaire (SF-36) [2]. This is particularly true for the physical functioning, physical role and vitality domains. Positive correlations have been observed between PF and both physical and mental health-related factors in the general population [3-5] and the elderly [2,6,7]. An approach based on the study of relationships between each HRQoL dimension and a multidimensional PF can contribute to identify the disaggregated usefulness of each PF tests to assess health-related quality of life adjusted by age and HRQoL. Additionally, it remains unclear how a low score in a specific PF component is associated with self-perception of problems in a specific HRQoL dimension. The Knowledge of these relationships will help to identify the PF components that most significantly limit the HRQoL of each individual by age, and this information might improve the specific group and individual-tailored exercise prescription in a more suitable manner.

Most previous studies have only evaluated PF by using aerobic endurance or strength measures [2,4,5,7,8] and thus, studies examining the associations between HRQoL and other PF components such as flexibility, balance, and agility are needed. In addition, most of the previous studies used the SF-36 to measure the HRQoL [2,7,9]; there are no studies that use the EuroQol 3 level version questionnaire (EQ-5D-3L), despite the fact that this is one of the most widely used HRQoL questionnaires due to its brevity, ease-of-use, and value in population and health economics analyses [10].

The analysis of these relationships between PF and HRQoL through the perception of absence or presence of problems for each HRQoL dimension would be the next step aimed to help detecting improvement needs of PF components based on self-perception of problems in HRQoL dimensions, as an alternative to traditional percentiles scores relating to age.

One of the major targets of public health programs is to increase PA levels in general population and specifically in those with lower levels. However, the previous studies that assessed PA or PF by objective methods, particularly those examining middle-aged and older adults, usually recruited a high percentage of participants who were physically active [11-13]. By contrast, the general population is mostly inactive [14-17]. Thus, while these studies show that the level of PA relates closely to the PF level and HRQOL, these results should be taken with caution when considering people who are physically inactive. Therefore, it is important to make an effort to recruit a representative sample in which inactive people are present in similar proportions as in the general population.

Consistently, the aim of study was to identify the PF tests of a multi-dimensional PF battery more related the perception of problems in each of the HRQoL dimensions assessed by EQ-5D-3L in a general community-dwelling middle-aged and older adults

## Methods

### Study design

The present cross-sectional study was conducted in the region of Extremadura, Spain. The regional government divides this large region into eight areas for the purposes of healthcare administration. Each area is demarcated according to geographical and demographic factors and the study sample was stratified according to the size of the population in each area.

In total, 37 assessors were recruited from sports sciences graduates who had had prior experience in assessing the fitness of older people and who were employed in the framework of the Exercise Looks After You (ELAY) program [18] (the tests were administered as part of their professional duties). This is a public health program that aims to promote PA for middle-aged and older adults. It is supported and managed by the Extremadura regional government and performed by the University of Extremadura.

All assessors received a testing manual that had been developed by the project managers and that described all the test procedures and protocols. In addition, all assessors completed a training course together. The course consisted of three 4-hour sessions and served to homogenize and standardize the assessment methods, thereby reducing intra- and inter-tester errors. Data of a test-retest reliability has been published in a previous article with the PF scores of participants in ELAY program [19].

All assessments were conducted at centers for senior citizens in a large indoor area such as a multipurpose room or gymnasium. The testing stations were distributed in 124 municipalities. Each tester administered the full battery of tests on a single day in the testing stations that were assigned. The participants were assessed separately and were instructed to wear appropriate clothing and footwear, to eat a light meal approximately 1 hour before testing, to avoid drinking alcoholic beverages in the preceding 24 hours, and to not perform vigorous PA the day before the assessment. In terms of testing safety, all participants were screened by using the Physical Activities Readiness Questionnaire (PAR-Q) and the resting blood pressure was checked to rule out those with cardiac illness or uncontrolled hypertension. Those who answered "yes" to any question on the PAR-Q or who had a blood pressure greater than 160/100 mmHg were excluded from the study.

### Participants

All participants (6243 females and 861 males aged 50-99 years) were community-dwelling individuals who had been recruited in the framework of the ELAY program.

To obtain a suitably wide spectrum of people in the study population, thereby reducing the risk of bias, the recruitment strategy centered on informing the public about the study and its objectives *via *several media: a) an initial announcement by the regional Health, Welfare and Culture and Sports ministers on mass media, namely the most relevant regional TV channels, radio stations, newspapers and web-sites; b) paid advertisements in these regional mass media for 3 months; c) announcements in the local mass media by 37 trained technicians; d) information (emails, center meetings, and printed brochures) that was disseminated to social workers and personnel working at primary care centers, nursing homes, and social centers for the elderly; e) posters and flyers addressed to the elderly that were attached on the walls of primary care centers, nursing homes, city halls, social centers for the elderly, sport centers, and local park entrances; and f) information stands at regional and local health-, sport- or welfare-related events (information meetings, fairs, etc.) for 1 year. The public health and sport program advertisements included the following information: a) the support of the study by the regional Health, Sport and Welfare ministries and the university; b) participants would not be required to pay a fee; c) participants would receive an individual health-related fitness report after undergoing a battery of tests and a lifestyle face-to-face interview; d) participants would undergo a short medical examination to ensure that they could walk in a group (see exclusion criteria below) and; e) participants would be offered a medical approval to participate in a supervised walk-based health-enhancing program. Although the percentage of people who were initially enrolled at primary care centers varied markedly between different practitioners depending on their willingness to recruit actively, the advertisements referred all volunteers to their primary care physician or nurse in the public sector to obtain physician approval. It is important to note here that all elder individuals are eligible for Spanish national healthcare and they do not have to pay a fee for primary health care consultations (apart from the taxes they pay to support the health care system). Therefore, there was no economic impediment to participate in the study.

Each volunteer was then assessed to see if he/she met the inclusion and exclusion criteria. This assessment was performed by primary health care personnel (general practitioner or nurse) who had comprehensive files on each volunteer. Eligible participants were those aged 50 and older who were functionally independent and could walk outside their house for 10 minutes without requiring help from another person. They also lacked medical conditions or physical or cognitive limitations that precluded their ability to follow instructions and participate safely in the battery of fitness tests and to complete questionnaires. The participation was voluntary and all subjects gave their written informed consent.

All protocols adhered to the updates of the Declaration of Helsinki, and the study was approved by the Committee on Biomedical Ethics of the University of Extremadura.

### Measurements

#### Demographic data

A general questionnaire that asked questions regarding age, marital status, educational level, smoking and alcohol habits, and the weekly level of PA was administered.

#### Health-related quality of life

The Spanish version of the EQ-5D-3L [20,21] was used to measure HRQoL. The EQ-5D-3L assesses five dimensions, namely mobility, self-care, usual activities, pain-discomfort, and anxiety-depression. The respondent is asked to indicate his/her health state in each of the five dimensions according to one of three levels: «no problems», «moderate problems», or «severe problems» [10]. This instrument is one of the most widely used HRQoL questionnaires due to its brevity, ease-of-use, and value in health economics analyses [10]. The responses in each dimension were also collapsed into a two-tier variable consisting of «perceive problems» and «do not perceive problems».

#### Physical Fitness

Each participant completed a multi-component battery of PF tests for middle-aged and older adults. The duration of this battery was approximately 45 min. Standardized instructions were given to all participants concerning the performance of the tests, namely that they should do their best but never overexert themselves or go beyond what they feel is safe for them personally. Weight and height were measured according to the recommendations of the European Council [22] for the calculation of body mass index (BMI). The test-battery was preceded by a series of general warm-up exercises involving 3 min of low intensity walking and stretching exercises of the lower and upper-body. The following fitness outcomes were then measured:

##### Upper body strength

Bi-handgrip strength was measured by using a grip-strength dynamometer (TKK 5401 Model). Two measures were taken for each hand and the sum of the maximal strength of each hand was recorded [23]. In a previous study of Spanish adults, the reliability coefficient for this test was ICC = 0.99 [24].

##### Upper and lower body flexibility

Upper body flexibility was measured using the back scratch test [25]. In this test, overlap between the fingers is scored positively and distance between the fingers is scored negatively. This test has a reported reliability of ICC = 0.96 [26]. Lower body flexibility was measured by using the seated sit-and-reach test of Jones *et al*. [27]. Two trials were performed for each side. The maximal score (right or left) of the two trials was recorded. This test has a reported reliability of ICC = 0.95 [26].

##### Balance and agility

Agility and dynamic balance were measured by using the 3 meter version of the Timed Up-and-Go Test (TUG) [28]. Static balance was measured by using the Functional Reach Test (FR) [29]. For each test, the best score of two trials was used. The FR has a reported reliability of ICC = 0.81 [29], and the TUG has a reported reliability of ICC = 0.98 [28].

##### Aerobic endurance

This was measured by using the 6.min walk test. This determines the maximum distance in meters that can be walked along a 20 meter corridor in 6 min [25]. Each participant performed only one trial of this test. This test has reported a reliability of ICC = 0.94 [26].

### Procedures

The participants did a general warm-up exercise consisting of 3 min of easy walking and easy lower- and upper-body stretching exercises. Immediately afterwards, the volunteers were all instructed as follows: "do the best that you can in the tests but do not push yourself to the point of overexertion or beyond what you believe is safe for you". In accordance with the recommendations of Rikli and Jones [11], for all tests except the 6-min walking test, the participants were asked to complete two trials, as this would allow the participant to become familiar with the test procedures. To minimize the effects of fatigue, the tests were administered in the following order: functional reach, chair sit-and-reach, TUG, handgrip, and back scratch. After a 5-min break, the 6-min walking test was administered.

### Data Analysis

The descriptive statistics are presented as frequencies and percentages. Correlation coefficients for the relationships between the EQ-5D-3L dimensions (Phi correlation coefficient) and the relationships between the PF tests (Pearson r correlation coefficient) were calculated. The correlation coefficients for the relationships between EQ-5D-3L dimensions and the PF tests were also calculated (Spearman's rho correlation coefficient). Two logistic regression models were developed for every EQ-5D-3L dimension, namely an adjusted model and an unadjusted model. Before conduct the modeling, the three levels of each dimension were collapsed into two levels: "no problems" and "moderate and severe problems". The latter served as the reference level. The unadjusted models only included PF test performances as predictors, whereas for the adjusted models, the following potential confounding factors were included: age, gender, weekly level of PA, smoking and alcohol habits, BMI, and educational level. Age and BMI was included in the analysis as continuous confounders, whilst rest of confounders was included according with the categories exposed in table 1. The cut-off points of probability that were used for logistic modeling were previously determined by a Receiver Operating Characteristics (ROC) analysis according to sensitivity-specificity pairs that maximized the Youden index. For each model, the odds ratios (ORs) linked to PF predictors and their respective significances were determined and the Hosmer and Lemeshow test for goodness of fit assessment of models was performed. Statistical analyses were performed with the SPSS 19.0 statistical software package and an alpha level of p < 0.05 was used for significance.

**Table 1:** Participant Demographics (N = 7104, 6243 females and 861 males)

	All (%)	Male (%)	Female (%)
**Age**			
50-54	171 (2.4)	4 (0.5)	167 (2.7)
55-59	534 (7.5)	24 (2.9)	510 (8.2)
60-64	1506 (21.2)	105 (12.2)	1401 (22.4)
65-69	1786 (25.1)	226 (26.0)	1560 (24.9)
70-74	1677 (23.7)	235 (27.4)	1442 (23.1)
75-79	970 (13.6)	160 (18.7)	810 (13.0)
80 +	460 (6.5)	107 (12.3)	353 (5.7)
All	7104	861	6243

**Marital status**			
Single	297 (4.2)	54 (6.3)	243 (3.9)
Married	4616 (65)	693 (80.5)	3923 (62.8)
Widow/separated/divorced	2142 (30.2)	111 (12.9)	2031 (32.5)
All	7055 (99.3)	858 (99.7)	6197 (99.3)
Missing	49 (0.7)	3 (0.3)	46 (0.7)

**Education**			
No formal education	4307 (60.6)	477 (55.4)	3830 (61.3)
Primary education	2464 (34.7)	314 (36.5)	2150 (34.4)
Secondary education	210 (3.0)	45 (5.2)	165 (2.6)
Superior studies	114 (1.6)	24 (2.8)	90 (1.4)
All	7095 (99.9)	860 (99.9)	6235 (99.9)
Missing	9 (0.1)	1 (0.1)	8 (0.1)

**Physical Activity at week**			
0 hours/week	3935 (55.4)	522 (60.6)	3413 (54.7)
< 3 hours/week	1533 (21.6)	149 (17.3)	1384 (22.2)
≥ 3 hours/week	1462 (20.6)	167 (19.4)	1295 (20.7)
All	6930 (97.6)	838 (97.3)	6092 (97.6)
Missing	174 (2.4)	23 (2.7)	151 (2.4)

**Smoking**			
+24 cigarettes/day	26 (0.4)	12 (1.4)	14 (0.2)
15-24 cigarettes/day	72 (1.0)	27 (3.1)	45 (0.7)
5-14 cigarettes/day	98 (1.4)	31 (3.6)	67 (1.1)
1-4 cigarettes/day	55 (0.8)	26 (3.0)	29 (0.5)
No smoke	6853 (96.5)	765 (88.9)	6088 (97.5)

**Alcohol habits (more than 1 glass/day)**			
Every day more than 1 glass	162 (2.3)	128 (14.9)	34 (0.5)
2-3 times/week more than 1 glass/day	113 (1.6)	64 (7.4)	49 (0.8)
1 time/week more than 1 glass/day	150 (2.1)	55 (6.4)	95 (1.5)
2-3 times/month more than 1 glass/day	284 (4.0)	34 (3.9)	250 (4.0)
Less than 1 time/month more than 1 glass/day	247 (3.5)	34 (3.9)	213 (3.4)
Never more than 1 glass/day	555 (7.8)	138 (16.0)	417 (6.7)
Never drink alcohol	5593(78.7)	408 (47.4)	5185 (83.1)

## Results

The demographic characteristics of the study participants are shown in Table 1. A total of 7104 individuals (6243 women and 861 men) aged 50-99 years were assessed. Individuals from the 60-79 years age group accounted for approximately 83.6% of the total study population. Most of the study participants were either married (65%) or widowed (28.3% of the 30.2% of individuals in the widowed/separated/divorced category). Most participants had not received formal education (60.6%) or had received only primary education (34.7%). Only 20.6% engaged in 3 or more hours of PA per week apart from that associated with the performance of daily living tasks such as shopping and cooking. The sample therefore comprised predominantly of participants who did not have regular formal PA. A high gender-specific difference in alcohol consumption was observed (87.1% of females were abstinent *vs*. 47.4% of males). The majority of both males and females were non-smokers (88.9% of males *vs*. 97.5% of females).

Correlation coefficients between all variables are shown in Table 2. There were significant correlations between all variables except anxiety/depression and the back scratch test. With regard to correlations between the five HRQoL dimensions, the dimensions that correlated best each other were mobility, self-care and usual activities, especially the variables pair self-care and usual activities. The PF tests that correlated best with the HRQoL dimensions were the TUG and the 6-min walk. The HRQoL dimensions that correlated least well with the PF tests were pain/discomfort and anxiety/depression. All PF tests were interrelated, especially the TUG and the 6-min walk.

**Table 2:** Correlation coefficients between Health-Related Quality of Life and physical fitness tests.

	Health-Related Quality of Life dimensions					Physical fitness tests					
	
	Mobility	SelfCare	Usual activities	Pain/Disconfort	Anxiety/Depression	Hand grip	Back scratch	Sit and reach	Functional reach	Time up & go	6 min walk
**Health-Related Quality of Life dimensions**											
Mobility	1.000										
SelfCare	0.317**	1.000									
Usual activities	0.397**	0.499**	1.000								
Pain/Disconfort	0.276**	0.141**	0.212**	1.000							
Anxiety/Depression	0.138**	0.119**	0.170**	0.235**	1.000						
**Physical fitness tests**											
Hand grip	-0.124**	-0.119**	-0.176**	-0.142**	-0.135**	1.000					
Back scratch	-0.145**	-0.124**	-0.166**	-0.085**	-0.014	0.062**	1.000				
Sit and reach	-0.120**	-0.169**	-0.169**	-0.082**	-0.073**	0.064**	0.279**	1.000			
Functional reach	-0.166**	-0.169**	-0.201**	-0.068**	-0.042**	0.266**	0.279**	0.316**	1.000		
Time up & go	0.254**	0.197**	0.258**	0.166**	0.157**	-0.288**	-0.317**	-0.292**	-0.398**	1.000	
6 min walk	-0.262**	-0.188**	-0.230**	-0.156**	-0.103**	0.291**	0.304**	0.245**	0.347**	-0.573**	1.000

Phi correlation coefficients between Health-Related Quality of Life dimensions

Spearman's rho correlation coefficients between Health-Related Quality of Life dimensions and Physical fitness tests.

Pearson r correlation coefficients between Physical fitness tests.

* p < 0.05; ** p < 0.001

The unadjusted and adjusted ORs obtained for each HRQoL dimension, their respective 95% confidence intervals (CI), and their *p *values are shown in Table 3. The unadjusted models only showed significant goodness of fit for the mobility and pain/discomfort dimensions. The adjusted models showed significant goodness of fit for all dimensions when the potential confounding variables (age, gender, weekly PA, smoking and alcohol habits, BMI and educational level) were included. The inclusion of potential confounding variables in the model did not change the significance of the ORs of the main variables in each dimension, except for the back scratch test with regard to the mobility dimension. For both the unadjusted and adjusted models, the highest ORs for each dimension were for the TUG.

**Table 3:** Odds ratios (ORs) and 95% confidence intervals (CI) for perceived problems in each EQ-5D-3L dimensions and physical fitness tests

Target	Predictors	Unadjusted model			Adjusted model^a^		
		
		OR (95% CI)	*p*	Goodness of fit	OR (95% CI)	*p*	Goodness of fit
MO	Hand grip	1.00 (0.99-1.00)	0.07	Χ^2 ^= 14.57 **p = 0.07**	1.00 (0.99-1.00)	0.21	Χ^2 ^= 5.67 **p = 0.68**
	Back scratch	0.99 (0.99-1.00)	**0.03**		1.00 (0.99-1.00)	0.15	
	Sit and reach	1.00 (0.99-1.01)	0.94		1.01 (1.00-1.01)	0.08	
	Functional reach	0.99 (0.98-1.00)	0.08		0.99 (0.98-1.00)	0.10	
	Time up & go	1.09 (1.06-1.12)	**< 0.01**		1.08 (1.05-1.11)	**< 0.01**	
	6 min walk	1.00 (0.99-1.00)	**< 0.01**		1.00 (0.99-1.00)	**< 0.01**	

SC	Hand grip	0.99 (0.98-1.00)	**0.01**	Χ^2 ^= 25.67 p = < 0.01	0.98 (0.97-0.99)	**< 0.01**	Χ^2 ^= 13.18 **p = 0.11**
	Back scratch	0.98 (0.97-0.99)	**< 0.01**		0.98 (0.97-0.99)	**< 0.01**	
	Sit and reach	0.99 (0.99-1.00)	0.10		1.00 (0.99-1.01)	0.59	
	Functional reach	0.98 (0.97-0.99)	**0.01**		0.98 (0.97-1.00)	**0.05**	
	Time up & go	1.07 (1.03-1.11)	**< 0.01**		1.06 (1.02-1.10)	**< 0.01**	
	6 min walk	1.00 (0.99-1.00)	**< 0.01**		1.00 (1.00-1.00)	**< 0.01**	

UA	Hand grip	0.98 (0.97-0.99)	**< 0.01**	Χ^2 ^= 17.71 p = 0.02	0.98 (0.97-0.99)	**< 0.01**	Χ^2 ^= 4.76 **p = 0.79**
	Back scratch	0.98 (0.98-0.99)	**< 0.01**		0.99 (0.98-0.99)	**< 0.01**	
	Sit and reach	0.99 (0.98-1.00)	**< 0.01**		0.99 (0.99-1.00)	**0.02**	
	Functional reach	0.98 (0.97-0.99)	**< 0.01**		0.99 (0.97-1.00)	**0.01**	
	Time up & go	1.10 (1.07-1.13)	**< 0.01**		1.09 (1.06-1.13)	**< 0.01**	
	6 min walk	1.00 (1.00-1.00)	**< 0.01**		1.00 (1.00-1.00)	**< 0.01**	

P/D	Hand grip	0.98 (0.98-0.99)	**< 0.01**	Χ^2 ^= 13.26 **p = 0.10**	0.99 (0.98-0.99)	**< 0.01**	Χ^2 ^= 11.18 **p = 0.19**
	Back scratch	0.99 (0.99-1.00)	**< 0.01**		0.99 (0.98-1.00)	**< 0.01**	
	Sit and reach	1.00 (0.99-1.00)	0.15		1.00 (0.99-1.00)	0.09	
	Functional reach	1.02 (1.01-1.03)	**< 0.01**		1.01 (1.01-1.02)	**< 0.01**	
	Time up & go	1.10 (1.06-1.13)	**< 0.01**		1.10 (1.07-1.14)	**< 0.01**	
	6 min walk	1.00 (1.00-1.00)	**< 0.01**		1.00 (1.00-1.00)	**< 0.01**	

A/D	Hand grip	0.98 (0.97-0.98)	**< 0.01**	Χ^2 ^= 15.92 p = 0.04	0.98 (0.98-0.99)	**< 0.01**	Χ^2 ^= 11.71 **p = 0.16**
	Back scratch	1.00 (0.99-1.00)	0.22		1.00 (0.99-1.00)	0.17	
	Sit and reach	1.00 (1.00-1.01)	0.15		1.00 (0.99-1.00)	0.79	
	Functional reach	1.00 (1.00-1.02)	**< 0.01**		1.01 (1.00-1.02)	**0.02**	
	Time up & go	1.00 (1.07-1.13)	**< 0.01**		1.12 (1.09-1.15)	**< 0.01**	
	6 min walk	1.00 (1.00-1.00)	0.55		1.00 (1.00-1.00)	0.46	

MO: Mobility; SC: Self-care; UA: Usual activities; P/D: Pain/Discomfort; A/D: Anxiety/Depression.

^a^Adjusted for: age, gender, weekly level of physical activity, smoking and alcohol habits, Body Mass Index and educational level.

## Discussion

The main finding of this study was that performance in a PF test battery that assess several fitness components could predict the self-reported perception of problems in the established HRQoL dimensions that are measured by EQ-5D-3L. These results were observed with a large cohort of community-dwelling middle-aged and older adults. The association between the PF tests and HRQoL was particularly strong for the physical HRQoL dimensions, namely the mobility, the self-care, and the usual activities dimensions; the latter two dimensions also relate to mobility-related activities. This finding agrees with the results of previous studies that observed associations between physical HRQoL dimensions and PF but also observed a slight association between mental health-related HRQoL dimensions and PF [2,6-9]. Most of these studies evaluated HRQoL by using the SF-36 and to our knowledge, this is the first study where the EQ-5D-3L questionnaire was used to evaluate the association between fitness performance and HRQoL.

The correlations between the HRQoL dimensions showed reveal a pattern of associations that allows us to group the mobility, the self-care, and the usual activities dimensions together. This reflects the fact that they all refer to physical function. This group is consistent and more suitable for comparisons with the results gathered by other HRQoL instruments such as the SF-36 that specifically calculate a physical component score (such comparisons are generally hampered because of the methodological differences between the questionnaires in terms of scale, valuation, and other factors). Indeed, a previous study has shown that the mobility, the self-care, and the usual activities dimensions of EQ-5D-3L correlate more closely with the physical component of the Short-Form 12-Item Health Survey (SF-12) (a shorter version of SF-36) than with its mental component [30].

The correlations between the PF tests also showed that the functional reach test, the TUG, and the 6-min walk could be grouped; this reflects the fact that they all relate to mobility-related activities. Supporting this is that the higher correlations were obtained between the three physical function-related HRQoL dimensions and these three mobility-related PF tests. PF correlated less well with the anxiety/depression and pain/discomfort HRQoL dimensions; however, of all PF tests, the ones that related better with these dimensions were the handgrip test, the TUG, and the 6-min walk. This supports the notion that PF tests, especially those that measure mobility-related activities, correlate better with the HRQoL dimensions that relate to the physical component, although small associations between PF and the mental dimensions of HRQoL were also observed.

In the present study, logistic regression model analysis revealed a statistically significant association between PF and the mental domains of HRQoL. Similar analyses performed by Takata *et al*. [7] and Wanderley *et al*. [2] did not observe this association. This discrepancy may be explained by disparities in sample size: the present study had 7104 subjects while the latter studies had 207 and 85 participants, respectively. Sample size is known to strongly influence statistical significance: a larger sample size leads to more accurate parameter estimates, which increases the ability to find significant associations.

Although the current study observed significant associations between PF components and HRQoL dimensions, the respective ORs were not large enough to obtain a significant goodness of fit in the unadjusted models (except for the pain/discomfort dimension). However, the adjusted models, where potential confounding variables had been included, showed a good fit. This is consistent with previous studies that demonstrated that HRQoL is influenced by BMI [31], education level [31], smoking habits [32], and gender [33], while fitness performance is influenced by PA [1], age and gender [11]. Therefore, the improved goodness of fit may be because the inclusion of these variables changed the relationships between the PF components and the HRQoL dimensions; alternatively, these variables had marked effects that are attributable to themselves. Either mechanism explains why we observed an effect of confounding variables on the fit of the models. Notably, however, the only cases where significant ORs became non-significant after adding confounding variables were the associations between the back scratch test and the mobility dimension, and between the functional reach test and the self-care dimension. The other tests remained stable after the addition of confounding variables. Hence, the improvement in the goodness of fit cannot be due to confounding effects, rather it reflects the main effects of these variables.

In the present study, the majority of the participants performed less than 3 hours of PA per week apart from that involved in the performance of daily living tasks so they do not have regular, formal physical activity. This is consistent with that reported by recent epidemiological studies in Spain [16,17]. By contrast, previous studies examining the relationship between HRQoL and PF had study cohorts where a high percentage of participants were physically active [2,6]. In another study, the PA of the participants was not reported [7]. The high proportion of physically inactive participants in the present study can be attributed in part to the recruitment strategy. The participants were recruited within the framework of a public health program for the elderly (institutionalized or community-based) that was supported by the regional government and with the implication of personnel working at primary care centers in the recruitment process. The personal contact with a general practitioner and the individualized nature of the program may also have been incentives for less physically active individuals who may otherwise have been unwilling to participate in any PA due to factors such as low self-confidence or limited interest in sport [34].

### Main strengths

Overall, the current study identified the fitness test more related to perceive problems in each dimension of HRQoL in a large sample of community-dwelling middle-aged and elderly population including a high percentage of participants who did not have regular formal PA as general population exhibits. This novelty could contribute to improve the efficacy and efficiency (e.g. selecting the most appropriate tests for monitoring and reporting needs and achievements related to HRQoL) in the assessment and monitoring of Health Enhancing Physical Activity programs.

### Limitations

The inclusion criteria of the present study ensured that all participants were able to walk at an easy pace for 10 min and to complete the socio-demographic questionnaire. It is therefore not possible to generalize the results to individuals with severe major physical disease or cognitive impairment. Further studies that analyze the relationships between PF and HRQoL in such populations are needed, although it should be noted that new adapted and validated tests are needed to measure PF in individuals with severe major physical disease.

In addition, studies that identify potential "cut-off points" in fitness performance that are associated with the presence of perceived problems in HRQoL dimensions are needed. Moreover, longitudinal studies that analyze the ability of a change in fitness performance to influence reported problems in HRQoL dimensions are required. Finally, studies that explore whether the EQ-5D-3L dimensions could be resume in physical and mental components are needed.

## Conclusions

The perception of problems in all of the EQ-5D-3L dimensions was associated with a lower level of fitness, particularly for those dimensions that relate more closely to physical components. The PF tests that associated most closely with the perception of problems in HRQoL dimensions were the TUG and the 6-min walk. This information will be useful for designing and assessing PA programs that aim to improve HRQoL.

## Competing interests

The authors declare that they have no competing interests.

## Authors' contributions

All authors contributed equally to this work. NG is the principal investigator of the ELAY study. All of the authors have read and approved the final manuscript.
